# Histone deacetylase inhibition: a novel approach to cancer treatment

**DOI:** 10.1038/sj.bjc.6603462

**Published:** 2006-12-12

**Authors:** B D Cheson

**Affiliations:** 1 Georgetown University Hospital, Lombardi Comprehensive Cancer Center, Washington, DC 20007-2197, USA

Lymphomas are a diverse group of malignant disorders that include non-Hodgkin's lymphoma and Hodgkin lymphoma. Collectively, lymphomas represent the fifth most common cancer type and the fifth leading cause of cancer death in the US. In 2006, the American Cancer Society estimates that more than 66 000 individuals in the US will be diagnosed with lymphoma, the majority of which (88%) will be classified as non-Hodgkin's lymphoma. Although enormous progress in cancer biology has been made over the past few decades, resulting in an overall decrease in the frequency of most cancers, the incidence of lymphomas continues to increase unabated. Clearly, an urgent need exists for newer and more effective treatments for these diseases. Far too many years have been spent combining existing cytotoxic drugs with only limited success.

In general terms, current cancer treatment strategies are tending to move away from nonspecific cytotoxic agents to biologically targeted drugs with more specific mechanisms of antitumour action. This approach is especially true for the management of non-Hodgkin's lymphoma, whose cancer cells can be targeted with greater reliability. One such group of targeted chemotherapeutic agents is the histone deacetylase (HDAC) inhibitors, which promote gene transcription by decreasing binding of histones to DNA and thereby produce growth arrest and apoptosis in cancer cells. Histone deacetylases have been implicated in the pathogenesis of lymphomas via regulation of the BCL-6 gene and, thus, their inhibition may provide a unique target for antilymphoma therapy.

One promising new weapon in our armamentarium to treat patients with lymphomas is the HDAC inhibitor vorinostat (suberoylanilide hydroxamic acid or SAHA). This supplement highlights our current understanding of the antitumour effects of vorinostat and reviews our clinical experience to date on the use of vorinostat in treating advanced haematological malignancies, including Hodgkin's disease and select subtypes of non-Hodgkin's lymphoma. The three articles comprising this supplement are based on individual presentations by eminent investigators at a symposium held in conjunction with the 9th International Conference on Malignant Lymphoma in Lugano, Switzerland, on 7 June 2005. Dr Victoria Richon presents a wealth of experimental data delineating the biological basis for the antitumour activity of vorinostat. Her comprehensive review indicates that vorinostat produces potent antiproliferative effects on a wide variety of transformed cells through the accumulation of acetylated proteins, including the core nucleosomal histones and other proteins. However, the therapeutic benefit of HDAC inhibitors may well extend beyond simple chromatin remodelling and may also involve effects on the acetylation of essential transcription factors such as BCL-6. Dr Owen O'Connor discusses the initial clinical experience with vorinostat, demonstrating potent antitumour efficacy and a favourable tolerability profile of oral and intravenous formulations in a variety of haematological malignancies. Finally, Dr Madeleine Duvic provides exciting new clinical data showing the benefits of vorinostat therapy in treating advanced, heavily pretreated malignant cutaneous T-cell lymphoma patients.

Collectively, these presentations provide encouraging data on the potential clinical efficacy of vorinostat as a single therapeutic modality in treating patients with lymphomas. Undoubtedly, however, the greatest benefit from this agent will be as part of multidrug regimens. The rational combination of vorinostat with agents that have complementary mechanisms of antitumour action may ultimately lead to optimisation of chemotherapy regimens for lymphoma patients and perhaps lead to a reduction in our dependence on the more toxic, nonspecific cytotoxic agents. Combination therapies incorporating vorinostat are likely to contribute greatly to our increased ability to cure patients with lymphomas and may herald a new era in cancer therapy.

